# Profound intellectual disability caused by homozygous TRAPPC9 pathogenic variant in a man from Malta

**DOI:** 10.1002/mgg3.1211

**Published:** 2020-03-11

**Authors:** Katelynn M. Wilton, Lauren B. Gunderson, Linda Hasadsri, Christopher P. Wood, Lisa A. Schimmenti

**Affiliations:** ^1^ Mayo Clinic Alix School of Medicine Medical Scientist Training Program Mayo Clinic Rochester MN USA; ^2^ Department of Clinical Genomics Mayo Clinic Rochester MN USA; ^3^ Department of Laboratory Medicine and Pathology Mayo Clinic Rochester MN USA; ^4^ Department of Radiology Mayo Clinic Rochester MN USA; ^5^ Department of Otorhinolaryngology Mayo Clinic Rochester MN USA

**Keywords:** autism spectrum disorder, genetic disease, IDT13, intellectual disability, MRT13, *TRAPPC9*

## Abstract

**Background:**

Intellectual disability is a complex multi‐faceted condition with diverse underlying etiologies. One rare form of intellectual disability is secondary to the loss of TRAPPC9, an activator of NF‐κB and a mediator of intracellular protein processing and trafficking. TRAPPC9 deficiency has been described in 48 patients with more than 15 pathologic variants.

**Method:**

Clinical evaluation, magnetic resonance imaging, and whole‐exome sequencing were used to characterize the underlying cause of absent speech, restricted/repetitive behaviors, and worsening behavioral outbursts in 27‐year‐old man from Malta.

**Results:**

Magnetic Resonance Imaging showed morphologic abnormalities, including global cerebral and cerebellar hypoplasia. Genetic analysis through Whole Exome Sequencing identified a homozygous deletion (c.568_574del) in *TRAPPC9* resulting in a frameshift, premature stop codon, and ultimately a truncated protein (p.Trp190Argfs*95). In this case, the pathogenic variant was homozygous, identified in both of the parents without known consanguinity.

**Conclusion:**

Given the phenotype and genotype consistent with a deficiency in TRAPPC9, it is likely that this patient represents a novel case of this rare genetic syndrome. Specifically, this case, in the context of 48 total reported patients, raises questions as to the geographic origin of the pathologic variant and optimal detection and therapeutic intervention for this condition.

## Graphical abstract

A man previously diagnosed with autism spectrum disorder secondary to intellectual disability, absent speech and repetitive behaviors was found to have homozygous pathogenic variants in TRAPPC9. In the context of the currently reported 48 known cases of TRAPPC9 pathogenic variants, his phenotype is consistent with previously reported phenotypes. Interestingly, it is found that, although the known pathogenic variants do not cluster in respect to location within the gene, the known cases all appears to be from a localized geographic area.

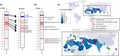

## 1 INTRODUCTION

Congenital intellectual disability can result from a variety of causes, including prenatal exposures, intrapartum complications, genetic variations, and idiopathic factors. Congenital causes can form stereotypical syndromic patterns, which can, in some cases, differentiate initial causes. However, in other cases, the congenital phenotype may not clearly indicate the underlying pathology. Given that critical early therapeutic interventions can help guide these individuals to more independent living, proper, and early diagnosis is essential.

Autism spectrum disorder is a broad diagnosis that encompasses social and language difficulties paired with repetitive and restricted interests (*Diagnostic and statistical manual of mental disorders: DSM‐5*, 2013). Autism spectrum disorder results from a complex mix of genetic and environmental factors and is currently treated with early intense therapy aimed at developing social and language skills, as well as targeting specific intellectual disabilities that can accompany the syndrome (Sacrey, Bennett, & Zwaigenbaum, 2015; Sanchack & Thomas, 2016; Sengupta, Lobo, & Krishnamurthy, 2017; Sharma, Gonda, & Tarazi, 2018; Tachibana et al., 2017). Children with this diagnosis who are treated early usually gain more developmental and social abilities, leading to greater independence later in life (Anderson, Liang, & Lord, 2014).

The recent rise of the genomic era has led to a great potential to improve the diagnosis and management of genetic disease. Many older patients diagnosed with autism spectrum disorder were diagnosed before the commonplace use of genetic testing or the discovery that many specific genes can be responsible for similar phenotypes. One such syndrome, currently called MRT13, arises from pathogenic variants in *TRAPPC9*, a protein known to increase activation of NK‐kB, and to aid the transport of vesicles from the rough endoplasmic reticulum to the golgi apparatus (Philippe et al., 2009; Zong et al., 2011, 2012). Twelve publications have detailed individuals with, generally deleterious, pathogenic variants in *TRAPPC9* that lead to a syndrome with severe intellectual disability, microcephaly, magnetic resonance imaging abnormalities, and in a majority of patients, dysmorphic facial features (Abbasi et al., 2017; Abou Jamra et al., 2011; Duerinckx et al., 2018; Giorgio et al., 2016; Hnoonual, Graidist, Kritsaneepaiboon, & Limprasert, 2019; Kakar et al., 2012; Koifman et al., 2010; Mir et al., 2009; Mochida et al., 2009; Mortreux et al., 2018; Najmabadi et al., 2007; Philippe et al., 2009). Most of these patient reports describe individuals who never gained the ability to verbally communicate, in some cases, despite intensive interventions.

Here, we report a 27‐year‐old man from Malta who was initially diagnosed with Autism Spectrum disorder due to repetitive stereotypic behaviors and absent speech at 18 months. He underwent interventions, including almost fifteen years of speech therapy, without ever developing speech. At age 27, he presented again for increasing agitation and outbursts, especially with increased stimulation. Whole exome sequencing revealed a 7 base pair deletion in *TRAPPC9* that resulted in a premature stop codon and truncation of the protein.

## 2 CASE PRESENTATION

**Figure 1:**
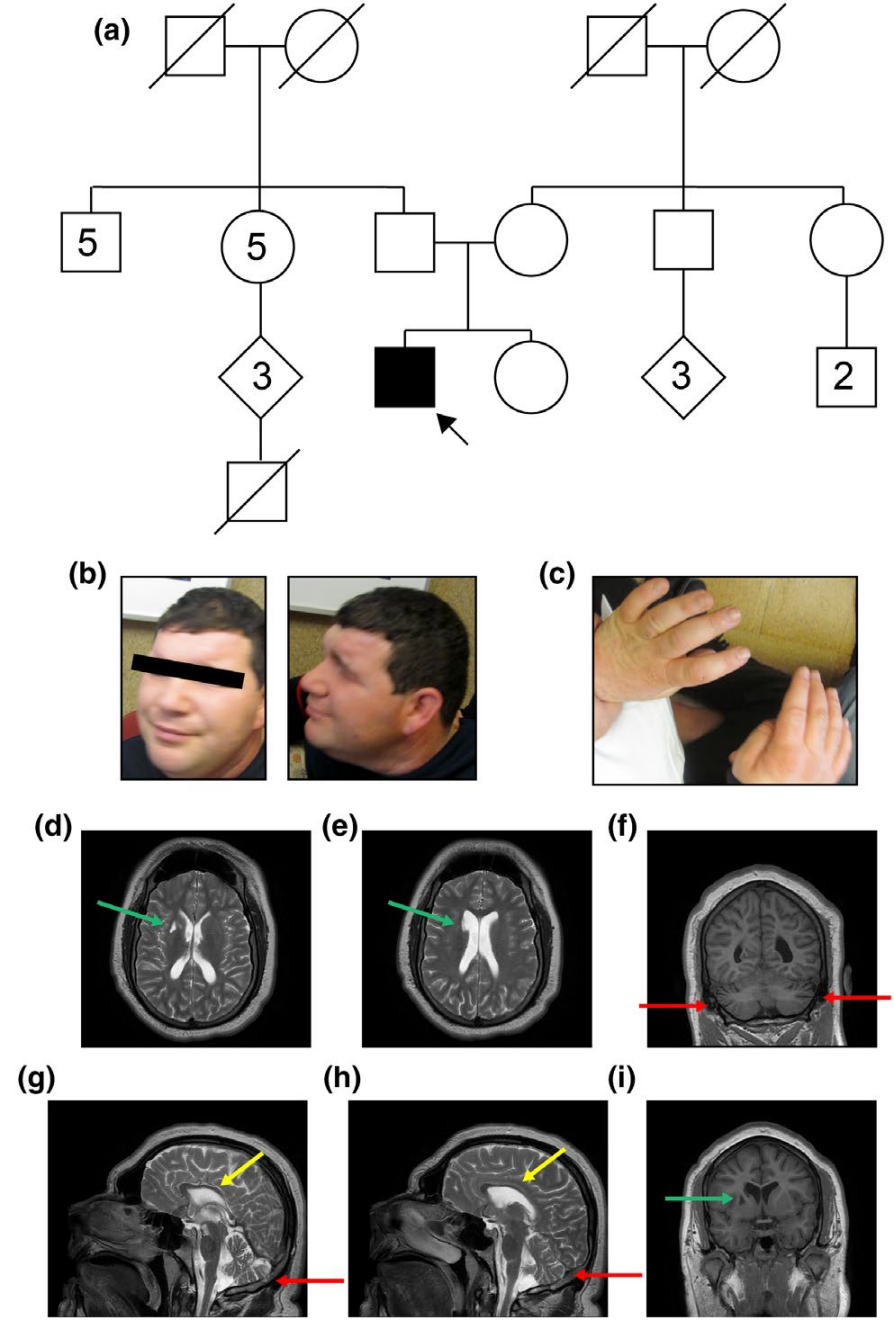
Phenotypic Findings in a 27‐year‐old man with IDT13 due to TRAPPC9 deficiency (a) Pedigree representation of the patient and 2 generations of his family. Patient is indicated by a black arrow. Boxes represent male family members. Circles represent female family members. Filled in shapes indicate the presence of autism, intellectual disability or both. (b) Photograph of facial and cranial features from front and side showing general microcephaly, sloping forehead, and prominent brow. (c) Photograph of hands from above, showing small dimensions, given patient height. (d–i) Magnetic resonance imaging of the head including (d, e) axial T2‐weighted images, (f, i) coronal T1‐weighted images and (g, h) sagittal T2‐weighted images. Red arrows designate cerebellar atrophy. Yellow arrows indicate atrophy and thinning of the corpus callosum. Green arrows show a lacunar infarct in the anterior right basal ganglia

The patient is a 27‐year‐old gentleman from Malta who was born via normal vaginal delivery to non‐consanguineous parents with no known family history of intellectual disability or autism spectrum disorder (Figure 1a). He sat upright at 6 months and began walking by 14 months. As an infant, he showed some social skills, including smiling and making eye contact. However, at 19 months, he still had absent speech without any attempt at mimicking linguistic movements of the tongue and/or lips. He additionally showed repetitive clapping behaviors. Secondary to these findings, paired with a normal karyotype, he was diagnosed with autism spectrum disorder and underwent intensive therapeutic intervention, including speech therapy until age 16. At age 27, he had not developed speech, though he could follow simple commands. He was not able to perform any of his activities of daily living independently. He could walk, reach for desired objects and could choose to be cooperative, but had increasing outbursts with arm thrashing and loud vocalization, especially in response to increased stimulation and unfamiliar settings. He additionally displayed restricted and repetitive behaviors throughout his life.

The patient was alert, but did not make eye contact and was unable to respond to questions. Some dysmorphic features were observed, including a sloping forehead, prominent brow and fleshy ears with grooves behind each ear lobe (Figure 1b). Given his height (171.8cm), he had microcephaly, with a head circumference of 54.5cm, putting him at approximately the third percentile for head circumference (Bushby, Cole, Matthews, & Goodship, 1992). His hands were also small (hand length:17 cm; middle finger length:7cm; Figure 1c).

## 3 IMAGING INVESTIGATIONS

Two magnetic resonance imaging studies were completed over a decade apart, with similar findings. Most notably, there was global intracranial cerebral volume loss relative to small head size (Figure 1d–i). There was additionally cerebellar atrophy, as shown in Figure 1f–h (red arrows) and atrophy and thinning of the corpus callosum (Figure 1g,h, yellow arrows). Of note, there was a lacunar infarct in the anterior basal ganglia (Figure 1d,e,i, green arrows), including the head of the caudate nucleus.

## 4 GENETIC INVESTIGATIONS

Genetic investigation was multi‐faceted. Pharmacogenomics testing and medication review did not identify any known Cytochrome p450 variants that might be responsible for the increasing agitation in response to psychotropic medication. Whole Exome Sequencing (WES) performed at Mayo Medical Laboratories using a custom capture reagent as per previously established protocols (Cousin et al., 2019) identified a homozygous deletion of 7 base pairs in chromosome 8, within the *TRAPPC9* gene (c.568_574del). This homozygous pathogenic variant resulted in a frameshift and a premature stop codon (p.Trp190Argfs*95) and is not present in the gnomAD database (https://gnomad.broadinstitute.org/gene/ENSG00000170043). This variant is considered likely pathogenic based on criteria from (Richards et al., 2015), PVS1, PM2, PP4. WES variants were prioritized for manual review based upon rarity (i.e., minor allele frequency less than 1% in population allele databases including Genome Aggregation Database, Exome Variant Server/Exome Sequencing Project, and 1,000 Genomes), inheritance pattern (variants fitting de novo, autosomal recessive or X‐linked recessive patterns), and association with germline diseases overlapping with the patient's clinical features. An investigation of potential candidate genes was also undertaken, prioritizing rare de novo variants in genes not known to be associated with underlying germline disease but possibly linked to relevant disease pathways. No additional likely causative nor possibly relevant variants were identified in this patient. The reported variants were confirmed by automated fluorescence dideoxy sequencing (Sanger sequencing). Parental samples were also analyzed by WES, with analysis specifically targeted toward those variants seen detected in the patient. The reported variants in the patient that were also found in a parent were confirmed via Sanger sequencing.

## 5 DISCUSSION

TRAPPC9 deficiency results in severe intellectual disability usually accompanied by absent or delayed speech, and microcephaly, with varied reports of stereotypic movements and dysmorphic features (Abbasi et al., 2017; Abou Jamra et al., 2011; Duerinckx et al., 2018; Giorgio et al., 2016; Hnoonual et al., 2019; Kakar et al., 2012; Koifman et al., 2010; Marangi et al., 2013; Mir et al., 2009; Mochida et al., 2009; Mortreux et al., 2018; Najmabadi et al., 2007; Philippe et al., 2009). In all cases where an MRI has been reported, MRI abnormalities were described. Most notably, thinning of the corpus callosum was most commonly noted (Abbasi et al., 2017; Abou Jamra et al., 2011; Duerinckx et al., 2018; Giorgio et al., 2016; Hnoonual et al., 2019; Kakar et al., 2012; Koifman et al., 2010; Marangi et al., 2013; Mir et al., 2009; Mochida et al., 2009; Mortreux et al., 2018; Najmabadi et al., 2007; Philippe et al., 2009). In this case, we report on a 27‐year‐old man who presented at 18 months of age with absent speech and developmental delay and was subsequently diagnosed with an autism spectrum disorder. He was subsequently managed with treatment optimized for autism spectrum disorder, but never developed speech or independence with activities of daily living. At age 27, after advancement in genomic diagnosis, a seven base pair deletion resulting in a frameshift pathogenic variant in *TRAPPC9* was found in his genome, which is associated with an intellectual disability syndrome. Specifically, autosomal recessive, pathogenic variants in TRAPPC9 are associated with a condition in OMIM (#613192) called Mental Retardation Type 13. In this current era, the term “Mental Retardation” is no longer acceptable as either a medical or social term for an individual with intellectual disability, thus we propose that this condition should be called either TRAPPC9 deficiency or Intellectual Disability Type 13 (IDT13) and will use this terminology from this point forward.

Intellectual disability due to IDT13 is a rare condition, with a total of 48 individuals from nineteen families reported worldwide. The details of these individuals, as published, is reported in Table S1. In short, all patients identified had intellectual disability, with varying phenotypes, including absent speech or speech delay (42/42 where it was reported), microcephaly (41/45 where it was reported) and MRI abnormalities (22/22 where it was reported) with 20 cases specifically mentioning thinning of the corpus callosum. A few less common reported symptoms included characteristic facies and repetitive stereotypic movements. Of note, only 9 of the 48 patients were noted to have behavior disorders, including tantrums or outbursts, though none specifically excluded that phenotype. Given this low percentage of reportedly affected individuals, it is likely that this is not specifically a part of the syndrome, but rather an attempt by the patient to communicate discomfort. Of note, 84% of the individuals and sixty‐nine percent of the families in which this syndrome was diagnosed had some level of consanguinity, which likely allowed this rare autosomal recessive variant to surface.

**Figure 2:**
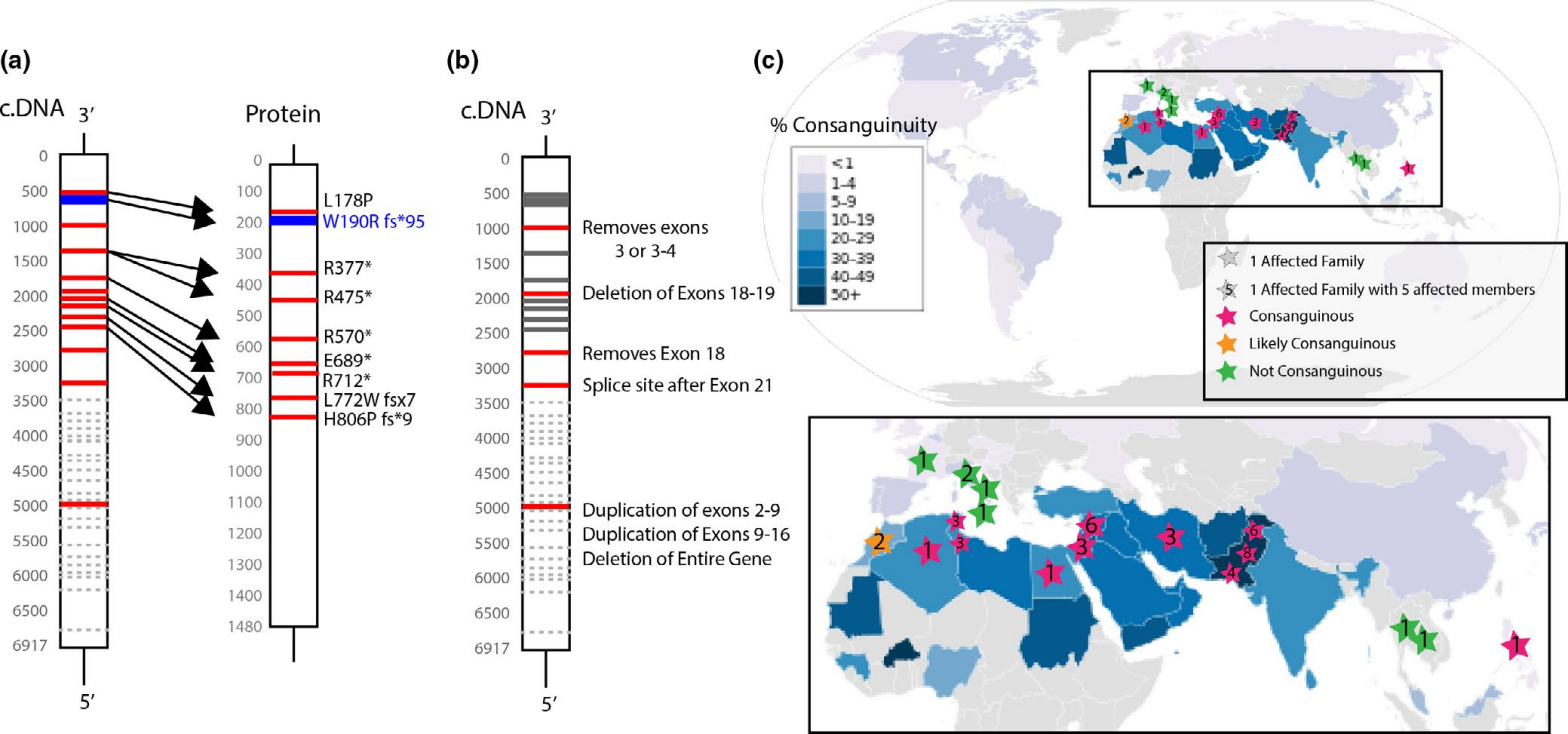
Pathogenic variants and Geographic Distribution of Reported Patients with TRAPPC9 deficiency or IDT13. (a) Reported gene and protein structures for reported simple pathogenic variants resulting in IDT13/ TRAPPC9 deficiency‐associated intellectual disability. Shown is the coding DNA (c.DNA) and the protein. Red bars indicate a previously reported pathogenic variant leading to IDT13. Blue bars indicate currently reported pathogenic variants. Dotted gray lines indicate mRNA sites where splicing to remove exons occurred. (b) Pathogenic variants reported to grossly alter the protein structure of TRAPPC9, pathogenic variants from (a) shown in gray. Citations for reported pathogenic variants can be found in Table S1. (c) Reported worldwide geographic country of origin or nationality as reported in published cases of TRAPPC9 pathogenic variants leading to IDT13. **Lower panel shows a** specific geographic area of origin where pathogenic TRAPPC9 pathogenic variants have been reported. Consanguinity map modified from (TheRedBurn, 2014) and used under Creative Commons Attribution‐Share Alike 3.0 Unported (https://creativecommons.org/licenses/by-sa/3.0/deed.en)

Within these 48 patients, there have been a number of pathogenic variants, most of which appear to result in decreased or absent TRAPPC9 protein (Figure 2a,b). The most common variant observed results in a truncated protein (p.Arg475X), which appears to be non‐functional and degraded (Abbasi et al., 2017; Abou Jamra et al., 2011; Giorgio et al., 2016; Mir et al., 2009; Mochida et al., 2009). In our case, we describe the second report of a seven base pair deletion (c.568‐574 del) which results in a premature stop codon and subsequent premature termination of the protein. In the previously reported case, this pathogenic variant was heterozygous with another deleterious variant (Mortreux et al., 2018).

Interestingly, the 48 reported patients with IDT13 congregate geographically in the Mediterranean area, including the Middle East, Southern Europe, and Southern Asia. As shown in Figure 2c, all reported families have originated from this region. Although this region does have genetically distinct traits, it is also an area of high consanguinity (Hamamy, 2012), which confounds the base genetic traits. Either of these factors could be responsible for the increased reports of IDT13 in this region.

The physiologic role of TRAPPC9 in intellectual capacity is still unclear. Specifically, TRAPPC9 is known to activate NF‐κB in neurons (Mir et al., 2009; Philippe et al., 2009), and to aid trafficking from the rough endoplasmic reticulum to the Golgi (Abbasi et al., 2017; Zong et al., 2011, 2012). It is mostly highly expressed in endocrine tissues and the brain (Berglund et al., 2008), where NF‐κB is needed for physiologic neuron and brain development (Crampton & O'Keeffe, 2013; Guerrini, Molteni, Wirth, Kistler, & Blasi, 1997; Widera, Mikenberg, Kaltschmidt, & Kaltschmidt, 2006; Zhang et al., 2012). As a result of this, under‐activation of the NF‐κB pathway during embryogenesis is thought to play a dominant role in the structural and phenotypic changes seen in IDT13. However, the action of TRAPPC9 in intracellular trafficking cannot be excluded, as NF‐κB trafficking is required for its effective activation (Mikenberg, Widera, Kaus, Kaltschmidt, & Kaltschmidt, 2006, 2007). Thus, it is possible that these two roles for TRAPPC9 may function in concert to promote effective neuronal NF‐κB function in neurogenesis. NF‐κB and TRAPPC9 are also expressed in a variety of other cells types, including adult neurons (Berglund et al., 2008). It is not known whether TRAPPC9 deficiency affects the function of adult neurons, or if the lack of the protein can cause accumulating damage that could be prevented.

Autism spectrum disorder and IDT13 have a number of overlapping phenotypes, though their core features vary considerably. A diagnosis of autism spectrum disorder is based on a deficiency in social skills and understanding paired with restricted interests and stereotypic movements. Specifically, the DSM 5 denotes that there must be “persistent deficits in social communication and social interaction across multiple contexts,” and “restrictive, repetitive patterns of behavior, interests or activities,” with symptoms first presenting in early development, and causing clinically significant impairment in functioning. In addition, social communication must be low even when accounting for a developmental level. In this case, the patient is not able to verbally communicate and only appears to communicate in reference to his needs, which sometimes occurs through violent outbursts. In addition, he did display repetitive and restricted behaviors since childhood. Given his severe deficits, he meets the criteria for a diagnosis of Austism Spectrum disorder with intellectual and language impairment secondary to TRAPPC9 deficiency and requires “very substantial support”, as designated by these criteria. Recent changes in the DSM 5 (*Diagnostic and statistical manual of mental disorders: DSM‐5*, 2013) have led to intellectual disability being removed as a core feature and instead of contributing to a portion of the spectrum. In contrast, IDT13 appears to have intellectual disability and speech impairment as core phenotypes, with stereotypic movements and social impairment being more varied. It is clear that severe versions of these two conditions can easily overlap, causing diagnosis to become difficult. In this case, an 18‐month‐old was diagnosed with an autism spectrum disorder and later found to also have TRAPPC9 deficiency/ IDT13. This may be a common circumstance—TRAPPC9 deficiency can masquerade as a complex and severe case of autism, causing its incidence to be highly underreported.

Proper identification and evaluation of individuals with an intellectual disability are critical. Like many of the other reported patients, the man described here has never developed speech, and fails to attempt normal linguistic movements, despite almost fifteen years of speech therapy. Given further research, and early identification of other children with this genotypic variant, a more optimal therapeutic regimen might be identified for these children. For instance, if speech therapy is not effective in helping these individuals to communicate, their time and resources might be better spent learning visual or habitual methods of communication or practicing motor skills to help with their activities of daily living. Depending on the prevalence of undiagnosed or misdiagnosed children with TRAPPC9 deficiency (IDT13), it may be possible to systematically study and optimize early therapeutic approaches.

## 6 CONCLUSION

Intellectual disability and autism spectrum disorder can present with a variety of phenotypes, and has been diagnosed in a large number of patients with intellectual and social disabilities. Given the heterogeneous nature of this disorder, the prevalence of genetic sub‐types of autism are likely confounded by general diagnoses that predate widespread genomic medicine. Given the unknown prevalence, needs and potential of individuals with specific genetic diseases related to autism, early identification of these cases may lead to optimized interventions that may or may not overlap with current autism therapies. Children with impaired speech and severe intellectual disability from the Mediterranean or Middle Eastern countries, especially with a consanguineous family history, should be considered for more intensive genetic testing.

## CONFLICT OF INTEREST

The authors have no conflicts of interest to declare.

## Supporting information

Table S1
